# Supplementary material for: Uncertainties in measuring offshore gambling: A scoping review of Nordic approaches
Source: PLoS One. 2026 Jan 23;21(1):e0340727. doi: 10.1371/journal.pone.0340727 (PMC12829935; doi:10.1371/journal.pone.0340727)
Supplement: Attachment — Submitted filename: response to reviewers.docx [file pone.0340727.s002.docx]

**Response to reviewers:**

Reviewer #1: Thank you very much for reading your article. The topic is very novel but I have serious doubts about the article.
1.Over-reliance on a single data source: Most of the data used in the study come from H2 Gambling Capital, with a substantial portion funded by the gambling industry. This dependence on industry-sponsored data introduces a potential conflict of interest, as these sources are likely to overestimate offshore gambling market size, aligning with industry interests. This reliance weakens the objectivity and credibility of the research findings.

- As this is not a systematic review but a scoping review, we feel this is not so much a problem as an outcome in the study. We have highlighted that this is a critical scoping review, not a systematic review in the beginning of the methods section. The aim is not to provide a synthesis but a critical evaluation of existing methodologies and estimates.

2.Insufficient discussion and control of bias: Although the article acknowledges the influence of industry funding, it does not adequately address or correct for these biases. For instance, data funded by the industry tend to show inflated offshore gambling estimates, yet there is limited comparative analysis or adjustment for this potential distortion in the study.

- The methods section now highlights the key difference between scoping reviews and systematic reviews: Based on Tricco et al., the former are ‘generally conducted to provide an overview of the existing evidence regardless of methodological quality or risk of bias- Therefore, the included sources of evidence are typically not critically appraised for scoping reviews.’. We have also highlighted how the range of methods used in included studies does not permit using a single tool for risk of bias assessment.

3.Methodological limitations: The scoping review approach is suitable for an exploratory synthesis of existing research but lacks the depth of empirical analysis and verification that SSCI-level research typically demands. The study does not incorporate rigorous statistical or econometric methods to address potential biases, nor does it apply strict criteria for selecting data sources.

- We believe this comment results from a misunderstanding. This paper follows a scoping review methodology, not a systematic review methodology. We have therefore not been able to use such methods.

4.Public policy and health impact analysis: This paper discusses the public health risks of overseas gambling and the industry's resistance to legislation, which has certain policy significance. But SSCI journals typically expect deeper empirical support or validation of policy recommendations through theoretical models.

- We have expanded the policy implications and provided detailed recommendations for the development of a compound measure in the discussion. We also expanded on the relevance on global governance using Castel’s theory.

5.Weak Theoretical Foundation
The article lacks a robust theoretical framework, with limited exploration of theories related to offshore gambling measurement and its connection to consumer behavior or policy-making. SCI journals typically expect a strong theoretical basis; thus, expanding the discussion to include relevant theories would provide a clearer foundation for the study.

- Thank you. There are no existing theoretical models in this field, which is also why this scoping review is so important to build such foundation.

6.Data Processing and Transparency Issues
The transparency of data processing is limited, especially regarding the treatment of gray literature and industry-sponsored data. Clearer criteria for selecting industry reports and detailed explanations of calculation methods are necessary to improve the study’s reproducibility and transparency.

- This is because industry reports are generally not very open about their methodologies. This is again is more a result than a methodological limitation, in our opinion.

7.Insufficient Discussion of Methodological Differences
The article applies different methods (such as population surveys and revenue estimates) for measuring offshore gambling but lacks an in-depth discussion on how these methods affect results. A comparative analysis of potential biases and limitations across different methods, especially between industry-sponsored and government data, would enhance the study’s credibility.

- We have added discussion on this in the results and discussion to the extent possible. As we write, methodologies could largely not be compared due to limited data and standardisastion in the field.

8.Limited Temporal and Sample Scope
While the article covers multiple countries, the timeframe and sample size are relatively narrow, restricting its ability to reveal long-term trends. Expanding the study’s temporal scope and sample size could offer a more comprehensive analysis of changes in offshore gambling over time.

- As this is a scoping review that aims at investigating different methods and diversity in the field,

Reviewer #2: Summary: The paper represents an exploratory study of the offshore gambling market. It is a scoping study and therefore is an attempt to introduce the available metrics to estimate a market which has so far received limited analysis. It provides an overview of the issues that need to be addressed to gather a better understanding of the phenomenon.

Assessment: Although I am not an expert in the field, I believe the paper barely scrapes the surface of addressing the measurement of offshore gambling markets. In my view this is not sufficient to qualify as a research article. Perhaps the paper would better qualify as a literature review that identifies the open issues that need to be addressed. In order to qualify as research paper, I believe the authors should devote more effort to thinking about a research design that can potentially produce a better measure of offshore gambling markets, by perhaps conducting a survey (or experiment) on potential gambling consumers to address the determinants of their choice of online platforms.

- This was probably a misunderstanding due to the submission system in Plos One. As the system does not include a separate category for systematic and scoping reviews, we had to submit this as a research paper. Obviously, as also stated in the title, this is a scoping review.

Major Comments

1) Giving a formal definition of offshore versus onshore markets could be useful at the beginning of the paper. In particular, the distinction between "grey" and "black" markets is not obvious and should be made at the beginning of the paper to clarify exposition.

- This is now in the first paragraph of the introduction.

2) The comparison between the channeling rates measured in GGR share versus population share is not very clear. The first measures the share of those using onshore markets while the latter seem to measure the share of those using the offshore market. It would be useful to have a consistent concept of channeling rate, so that a higher rate uniquely defines more or less onshoring. If I understood this correctly, the following statement on lines 262 -264:
“Overall, and similarly to GGR-based channelling rates, population-based channelling rates were higher in monopoly countries (Norway, Finland) and lower in license-based countries (Sweden).” is correct, but not obvious when comparing it to that on lines 227-229:

“Overall, estimates of channelling rate are lower in monopoly regimes (Finland, Norway, Sweden before 2019). The channelling rate is higher in licensing regimes (Denmark, Sweden after 2019). However, different methodologies yielded differing results.”

- This is completely true. We clarify the issue now in the beginning of the results section focusing on population prevalence. We have also revised the indicated sentence to be less confusing.

3) While measuring the size offshoring markets as a share of GGR is partially informative, a more complete measure should also include the total value of the offshore market, since as the authors mentioned in the discussion, a reduction on the total size of a gambling market can lead to a rise in the share of offshoring that could correspond to a reduction in the GGR of offshore markets. Relatedly, it is not obvious why monopoly markets should be characterized by more offshore gambling in absolute terms, since a better regulated monopoly could potentially lead to a reduction of the onshore market without necessarily impacting the offshore market.

- This was a really helpful comment. We have now included a new section in the results where we explore this issue in more detail. The section is based on GGR market data. Unfortunately, and as descriptive of the field of offshore measurement in general, we were only able to do this using H2 data. However, the limitations of this are discussed.

Minor comments & Typos

Line 95 : “..whom, AND what kind of..”
Line 163: the acronym GGR is introduced before being defined. It is only defined on line 172.

- Corrected, thank you.

Reviewer #3: Measuring offshore gambling: A scoping review of Nordic approaches

The manuscript addresses an important topic by systematically reviewing approaches to measuring offshore gambling in Nordic countries. The topic is relevant, particularly given the regulatory challenges posed by offshore markets and their impact on public health and policy. In particular, the use of a scoping review approach and inclusion of diverse sources provides a broad overview of the topic. Here are my suggestions for the authors as the authors revise the paper

Major Comments:
1. The manuscript raises an important topic, but it could benefit from a clearer identification of the research gap. While the authors emphasize the seriousness of offshore gambling and the importance of channeling consumption from offshore markets to regulated markets, the rationale for conducting this systematic review needs further development. Specifically, why is it critical to understand market sizes and measurement methodologies in the context of existing research? Additionally, the justification for focusing on Nordic countries requires more detailed arguments.

- We have expanded the discussion on the critical relevance of the topic and on the Nordic context in the introduction.

2. Almost all articles rely on H2 data, and while the manuscript acknowledges this reliance, it provides limited details on the data itself and how the estimates are derived. Providing more comprehensive information about the data's characteristics and estimation methods would enhance the credibility of the review.

- This is a good observation. Unfortunately, as we also describe more clearly in the paper, we have no precise details on the provenance of H2 figures. This is characteristic of the entire field, and in our opinion, one of the central results of our paper.

3. Conducting a quality assessment in line with PRISMA guidelines would add rigor.

- We have expanded discussion in the methods on why we were not able to do this. See also response to R1 above.

4. The authors suggest multi-method approaches but do not offer concrete examples of how such methods could be implemented.

- We added several paragraphs of text in the discussion to create a roadmap towards a compound indicator. We hope this could serve as an example on how a better measuring practice could be developed.

5. In the same vein, while the manuscript discusses policy implications, these could be expanded further. Specifically, how should governments balance the need for accurate measurement with industry collaboration? What practical suggestions emerge from the review findings? While the systematic review demonstrates the authors' efforts, the discussion and conclusion sections need further development. Currently, the recommendations are presented without fully integrating the insights gained from the scientific review.

- We believe that the central issue is not to collaborate with industry, but to make sure industry is legally mandated to provide data to allow independent measurement. International collaboration is the other key. We have expanded on these policy implications in the discussion.

Minor Comments:
1. While the inclusion and exclusion criteria were discussed, the manuscript does not specify how many articles were excluded based on each criterion. Providing this information would strengthen the methodological rigor of the review.

- We added these figures to the Prisma flow chart, thank you for noticing this slip.
