# Supplementary material for: Uncertainties in measuring offshore gambling: A scoping review of Nordic approaches
Source: PLoS One. 2026 Jan 23;21(1):e0340727. doi: 10.1371/journal.pone.0340727 (PMC12829935; doi:10.1371/journal.pone.0340727)
Supplement: Attachment — Submitted filename: responses to reviewers.docx [file pone.0340727.s003.docx]

Journal Requirements:

1. When submitting your revision, we need you to address these additional requirements.

Please ensure that your manuscript meets PLOS ONE's style requirements, including those for file naming. The PLOS ONE style templates can be found at

<https://journals.plos.org/plosone/s/file?id=wjVg/PLOSOne_formatting_sample_main_body.pdf> and

[https://journals.plos.org/plosone/s/file?id=ba62/PLOSOne_formatting_sample_title_authors_affiliations.pdf](https://track.editorialmanager.com/CL0/https:%2F%2Fjournals.plos.org%2Fplosone%2Fs%2Ffile%3Fid=ba62%2FPLOSOne_formatting_sample_title_authors_affiliations.pdf/1/010f0195f3880420-b0a28f87-74f6-4d29-a994-ff2759f8863e-000000/9WEkz2rHusBAiaBwGqbEoN53rm5vyjIqGiyQqg8k6a0=205)

- We have made sure that the manuscript follows these guidelines

2. Thank you for stating the following financial disclosure: [VM and TR received funding from the Finnish Ministry of Social Affairs and Health based on provisions of the Finnish Lotteries Act §52. SK is funded by the University of Aalborg. HW received funding from the Swedish Research Council for Health, Working Life, and Welfare (Forte, grant number 2023-00898)].

Please state what role the funders took in the study.  If the funders had no role, please state: ""The funders had no role in study design, data collection and analysis, decision to publish, or preparation of the manuscript.""

If this statement is not correct you must amend it as needed.

Please include this amended Role of Funder statement in your cover letter; we will change the online submission form on your behalf.

- Thank you, this is now in the cover letter.

3. In the online submission form, you indicated that [All data used in the scoping review are fully available from public sources and from the authors. Data from H2 gambling capital are only available under license. These data can be made available by the authors with permission from H2.].

All PLOS journals now require all data underlying the findings described in their manuscript to be freely available to other researchers, either 1. In a public repository, 2. Within the manuscript itself, or 3. Uploaded as supplementary information.

This policy applies to all data except where public deposition would breach compliance with the protocol approved by your research ethics board. If your data cannot be made publicly available for ethical or legal reasons (e.g., public availability would compromise patient privacy), **please explain your reasons on resubmission and your exemption request will be escalated for approval.**

- We have created an OSF repository in which we have added all the underlying reports for our scoping review.
- We have not been able to include the key informant interview transcription nor the H2 gambling capital data in this repository. As also explained in our cover letter, the reasons for this are the following:

**H2 data:** These data are available under license. The license conditions prohibit us from sharing these figures openly. The data can be made available upon permission from H2 but we cannot make them publicly available.

**Key informant interview data**: Key informants were not asked for permission to share their interview transcription data openly. We also promised key informants that any direct quotations would be verified with them before publications. We are therefore unable to share these transcripts as this would breach confidentiality with respondents. while we recognize ideals of transparency and data sharing, we are obliged to respect that participants have not consented to making transcripts of their interviews publicly available.

4. Please include captions for your Supporting Information files at the end of your manuscript, and update any in-text citations to match accordingly. Please see our Supporting Information guidelines for more information: [http://journals.plos.org/plosone/s/supporting-information](http://track.editorialmanager.com/CL0/http:%2F%2Fjournals.plos.org%2Fplosone%2Fs%2Fsupporting-information/1/010f0195f3880420-b0a28f87-74f6-4d29-a994-ff2759f8863e-000000/TtFdxRClEykHFQqhyvQrhBc5UHFWqK5nIxKMm17EgPU=205).

- We do not have supporting files for this submission.

[Note: HTML markup is below. Please do not edit.]

Reviewers' comments:

Reviewer's Responses to Questions

**Comments to the Author**

1. Is the manuscript technically sound, and do the data support the conclusions?

The manuscript must describe a technically sound piece of scientific research with data that supports the conclusions. Experiments must have been conducted rigorously, with appropriate controls, replication, and sample sizes. The conclusions must be drawn appropriately based on the data presented.

Reviewer #1: Partly

Reviewer #2: Partly

2. Has the statistical analysis been performed appropriately and rigorously?

Reviewer #1: No

Reviewer #2: Yes

3. Have the authors made all data underlying the findings in their manuscript fully available?

The [PLOS Data policy](http://track.editorialmanager.com/CL0/http:%2F%2Fwww.plosone.org%2Fstatic%2Fpolicies.action%23sharing/1/010f0195f3880420-b0a28f87-74f6-4d29-a994-ff2759f8863e-000000/0jGkL6pZE0mbOMzvKoIPLfS67-GEr50tmlfADle7C6Y=205) requires authors to make all data underlying the findings described in their manuscript fully available without restriction, with rare exception (please refer to the Data Availability Statement in the manuscript PDF file). The data should be provided as part of the manuscript or its supporting information, or deposited to a public repository. For example, in addition to summary statistics, the data points behind means, medians and variance measures should be available. If there are restrictions on publicly sharing data—e.g. participant privacy or use of data from a third party—those must be specified.

Reviewer #1: No

Reviewer #2: No

4. Is the manuscript presented in an intelligible fashion and written in standard English?

PLOS ONE does not copyedit accepted manuscripts, so the language in submitted articles must be clear, correct, and unambiguous. Any typographical or grammatical errors should be corrected at revision, so please note any specific errors here.

Reviewer #1: No

Reviewer #2: Yes

5. Review Comments to the Author

Please use the space provided to explain your answers to the questions above. You may also include additional comments for the author, including concerns about dual publication, research ethics, or publication ethics. (Please upload your review as an attachment if it exceeds 20,000 characters)

Reviewer #1: It's an interesting research topic. The authors have discussed the issues of regulations and the gambling market. However, there are some issues that need to be pointed out:

1. The description of offshore gambling is not clear enough. The first thing that must be clearly defined is whether and how offshore gambling is regulated, and what the difficulties are in regulating area.

- Thank you for this observation. We have added a sentence in the first paragraph explaining why offshore gambling regulation is challenging.

2. It is suggested that the authors can plot the path of the research method into a block diagram to better understand the research process.

- We added a clear description to the introduction of the steps taken. The research process is also described in the prisma flow chart.

3. In line 404 "Offshore gambling is therefore likely to be highly risky for consumers."—— From an unregulated perspective, the risks of offshore gambling may not be measurable. But from the perspective of social risk management, it is necessary to study the regulation of offshore gambling. It is suggested that the authors distinguish between the definition and measurement of risk in the study.

- Thanks, this sentence was indeed somewhat unclear. We have now reworded the paragraph, explaining what we mean by risk and how it can be measured (with references to population studies).

4. The references should be revised.

- We have revised the references.

Reviewer #2: The authors apply a scoping review of offshore gambling and reveal available estimates from academic and grey literature in Denmark, Finland, Norway, and Sweden. They show four key results: (1) policy changes in the related debates may be connected to the prevalence of published offshore estimates; (2) offshore consumption typically consists of the most harmful gambling products; (3) offshore market shares tended to be lower than government-produced figures; (4) no gold standard or one reliable method to measure offshore gambling reliably. It is an interesting approach to estimate hard-to-measurement through literature reviews by tracking databases and shares from the existing literature. There are some issues for consideration:

Related Study Section
Authors should consider adding a short section introducing the use of a scoping review to estimate the scope of the hard-to-measurement industry. Those studies need not be about offshore gambling but scoping reviews. This would help the audience to see the value of adopting a scoping review for this study. For instance, authors should justify using a scoping review rather than a meta-analysis in this section, not the limitation section.

- This was a good comment. We added a few sentences on this in the last paragraph of the introduction to elaborate.

Methodological clarity
The authors analyze the available datasets from their reviews. Table 1: Please provide more content for the national databases and websites in the scoping review for audiences unfamiliar with offshore gambling.

- Thanks for this excellent comment. We have added a column in Table 1 with descriptions of the resources.

The exclusion of black market shares from the estimates could represent a major hurdle to the final objective of measuring offshore gambling, that is improving public health policy, as stated in the introduction to the study. However, this issue does not appear in the discussion nor in the recommendations sections. As a scoping review’s purpose is to explore an issue and raise new questions for researchers to address, it would be pertinent to encourage analyses covering all forms of online gambling abroad.

- This is another very good point. We have added discussion on this to the recommendations

As the estimates significantly differ between industry- and government-funded reports, explicitly comparing their methodologies more discriminately could reveal interesting patterns. This could be done not only in the text itself, but also visually in the figures.

- Thanks for this comment. We have redrawn the figures so that it is clear to see whether reports are publicly funded (red) or industry funded (black).

On the analysis :
Because the discrepancies between industry and government estimates are significant and have a political motivation, a discriminated analysis would be appropriate, as well as a differentiated visualization.

- We changed the figures as described above. We have also elaborated on these differences in the text.

It should be clarified why the H2 evolving estimates for 2023 are a problem. Adjusting predictions to the latest information is important for increasing estimate accuracy. The real issue is that we don't know what this new information is. That should be made explicit.

- Thanks. We agree that updates can also be viewed positively. We have elaborated on this point, explaining that this can be a problem because estimates are taken as snapshots for other reports and not revisited. The updates also create uncertainty for long-term use of these figures.

Bank data: one of the key informants suggests bank data as the best way to measure GGR and the authors are fully convinced. They include this strategy as a major point in their suggested compound measurement, but they don't discuss this idea in depth.
they say that only one study [27] uses bank data, but in the table study [29] is also listed as using bank data.
they should explain how bank data is used in the reports they mention, how it influences the estimates
they should discuss the drawbacks of banking data, such as issues of accessing the data, which is often protected.

- Thank you for flagging this. It was indeed study 29 (not 27) that mentioned banking data. This was a referencing error in the text.
- We also went back to the original source and the use of banking data is very vague. The report mentions using multiple data sources to estimate the size of the unregulated market. These include H2, company data, and estimates from the gambling regulator. The estimates from the gambling regulator are, in turn, based on multiple data sources including Norges Bank. What these data are and how they have been used is not elaborated. We were also not able to find details on the original regulator estimate. This is another example of how blurry most of these measures are (that are still promoted as the truth by lobbyists).
- As the use of bank data is this blurry, we decided that the best option was to remove the mention of bank data in the table and the text.
- Instead, we have expanded on the potential benefits and drawbacks of bank data in the discussion.

choice of countries: is it not too limiting for a scoping review? The explorative goal of such a study is to gather as many ideas and approaches as possible. Looking beyond these highly similar contexts with tight collaboration would be bring in more approaches to discuss.

- This is a valid point and something that we maybe did not elaborate on enough in the earlier version of the manuscript. We have now added a section on this in the first paragraph of the methods. We acknowledge that focusing on four countries, only, may be restrictive. However, since this question is highly contested and political, we feel that it is important to focus on contexts that we are also familiar with. This allows us to critically evaluate and understand underlying biases and connections of reports.

The exclusion of black market shares from the estimates could represent a major hurdle to the final objective of measuring offshore gambling, that is improving public health policy, as stated in the introduction to the study. However, this issue does not appear in the discussion nor in the recommendations sections. As a scoping review’s purpose is to explore an issue and raise new questions for researchers to address, it would be pertinent to encourage analyses covering all forms of online gambling abroad.

- This has been added to the recommendations. Thanks for a great point.

On the discussions and conclusions
Please state the estimated market size and types of channels of offshore gambling clearly in either the discussions or conclusions. Then, provide limitations of such estimation. Currently, the findings are weak and not focused.

- As explained in the introduction and the methods section, we did not produce a meta-analysis as this would have been tainted by very unreliable and potentially biased estimates. We feel that a meta-analysis would, in fact, do more harm than good in this case, at it would validate findings that may not be scientific. We also highlighted this again in the limitations.

Please use subtitles or highlights to make the findings clearer.

- We have added more subtitles and made them bigger for clarity (and following journal guidelines).

Minor comments:
-          On line 412 in the Discussion section, it is said that the “industry estimates of offshore market shares tended to be lower than government-produced figures”. This is inconsistent with the rest of the paper and is surely just a typing mistake.

- Thanks, we corrected this typo.

-      In the section called “Channeling rate by population share”, a sentence is formulated in a rather confusing manner: “Three studies, both from a country with a license-based system”. I would suggest “Three studies, among which two come from a country with a license-based system”.

- Thanks, we also corrected this sentence.

-          The last sentence before the Discussion section also lacks clarity. What

- Thank you, we fixed the sentence and it should now be clearer.
