# Supplementary material for: Uncertainties in measuring offshore gambling: A scoping review of Nordic approaches
Source: PLoS One. 2026 Jan 23;21(1):e0340727. doi: 10.1371/journal.pone.0340727 (PMC12829935; doi:10.1371/journal.pone.0340727)
Supplement: Attachment — Submitted filename: Responses to reviewers 2.docx [file pone.0340727.s004.docx]

Responses to reviewers

Thank you to the editors and reviewers for the time spent reviewing our manuscript. We appreciate all the comments, and have done our best to revise the manuscript accordingly.

Reviewers' comments:

Reviewer #3: General Comments
This is an interesting and timely study examining offshore gambling using a range of datasets. The topic is clearly important, and the use of multiple data sources strengthens the paper. However, there are several areas where the clarity of writing could be improved, particularly in the introduction and methods sections. A careful read-through is recommended to address grammatical issues throughout.

- Thank you! We have carefully reviewed the manuscript to fix the remaining grammatical issues and typos.

Introduction
• As this is a multidisciplinary journal, it’s important to clearly define key terms and contextualise the topic early on. Currently, the first half of paragraph one is somewhat confusing, particularly the explanation of offshore gambling. For instance, the statement “Any unlicensed offer can, in practice, constitute offshore gambling” is unclear. Does this mean that a gambling provider can be located within the same country as the gambler but still be considered offshore if unlicensed? If so, consider providing a concrete example to help readers unfamiliar with the regulatory context understand what this looks like in practice.

- This is an excellent point. We have revised the sentence.

• Lines 56–58: The sentence here is awkwardly worded. Please revise for greater clarity.

- Revised, thanks.

• Line 59: You mention that offshore offers increase competition in the market. It would be helpful to clarify whether this is being presented as a positive or negative effect, or whether it depends on context.

- Thank you for another excellent observation. We have explained how increased competition can have negative public health impacts.

• Line 98: Replace “on” with “across”.

- Done

• The final paragraph of the introduction currently includes justification for the scoping review methodology. Consider moving this to the methods section. You could then expand on the broader challenges with existing evidence in the field and clearly state the study’s aims in the final paragraph.

- A very good suggestion that we followed, thank you.

Methods
• You mention that online interviews were conducted, but there is no reference to ethics approval. Was ethics approval obtained, or was a waiver granted? This should be clearly stated.

- Good point, this has been included.

• The manuscript lacks detail on the data extraction process. Was this conducted by one author or multiple? Was a portion of the extracted data checked by a second author for accuracy? Please clarify.

- Included as well.

Results
• Line 248: You note that “key-informant interviewees highlighted the importance of multimethod analysis”, but do not elaborate on what was said to support this. While there is a quote a few sentences later, it’s not clear that this directly relates to the point. Consider clarifying or providing a more direct link between the interview data and this statement.

- Thank you. We chose to omit all direct quotations as some of our participants did not consent to us using these. Instead, we have expanded the descriptive summary.

• Line 373: Please define “skin betting,” as not all readers may be familiar with the term.

- Done

Discussion
• Lines 409–414 contain useful background information that would help to frame the paper. I suggest moving this content to the introduction to better set the scene and enhance interpretation of the results.

- Great idea, we have moved part of this to the introduction, but left the more detailed parts in the discussion as this detail is also a reflection of our results.

• Line 459: Replace “unclarities” with “ambiguities”.

- Done, thanks.

• Line 500: The suggestion that banks could provide access to transaction data seems unlikely. Blocking transactions is very different from sharing data. Has this occurred with other sectors or products? It may be worth noting that this would likely require government regulation to be feasible.

- Yes, you are correct. We have expanded on this.

• Line 506: Your third point also relies on voluntary collaboration. Historically, private providers rarely share data without a legal mandate. Consider acknowledging this limitation more directly.

- Yes, we have explicitly stated that this should be legally mandated, we did not mean for this to sound like we advocate for voluntary collaboration.

Reviewer #4: Premise of research study is focused on public health harm, however, authors do not acknowledge the greater public policy impact of online gambling for the legal-social aspect of data privacy, cybersecurity, and data security impact. May recommend minor revisions to acknowledge this in the project description and future steps/recommendations for frameworks for analysis.

- A very good suggestion, thank you! We have expanded on this in the introduction and the discussion.

Reviewer #5: This topic is very interesting, but I still have some questions:
1. First of all, the conclusion of this review relies on the screening of the literature, can you clarify the logic and basis of screening?

- We have expanded on the description of our screening methods in the methodology section.

2, for the main measurements of the comparison of conclusions, can use a table to make a clear show, such as common points or differences.

- This would be a great idea. Unfortunately, due to scarcity of data, we could not systematically compare measurements obtained using different methods or sources. This has been highlighted as an important step for further studies.

3, for the interview part, can make some basic introduction, the interview is what the population, whether it is representative, which is crucial to the conclusion of the impact.

- Thanks – we have expanded on the interviews in the methodology section.

4. Can the conclusions of the study be more specific in the abstract, or can some important conclusions be clearly shown?

- We added the main conclusion in the abstract.

Reviewer #6: I have carefully reviewed your research and have been deeply inspired by it. Here, I have several questions that I would like to discuss and exchange ideas with you further. I am well aware that some of these questions may not be entirely accurate, but I believe that through our communication, we can gain a deeper understanding of this subject. I look forward to your further response at your convenience. Thank you once again for your contributions to this field!

- Thank you so much for your comments and thoughts, we truly appreciate them and all the effort that you have put into reviewing our work!

1.Strengthen Term Definition: The operational distinctions between the “grey market” and the “black market” need to be clearly defined in the introduction, and differences in the Nordic regulatory frameworks should be supplemented (e.g., payment blockades in Finland/Norway vs. website blockades in Denmark).

- This has been added in the first paragraph of the introduction.

2.Explanation of Industry Report Motives: The potential motives for industry reports overestimating the offshore market need to be explained (e.g., lobbying for lower tax rates).

- Indeed. We cannot explain this with our data, but we can speculate – we have added some discussion on this in the discussion section.

3.Argument for Geographical Limitations: The study focuses on the four Nordic countries (Denmark, Finland, Norway, Sweden), but the representativeness for other jurisdictions has not been sufficiently argued.

- Another excellent point, we have added some discussion on this in the limitations section.

4.H2 Data Validation: The estimation methods of H2 Gambling Capital data need to be explained (e.g., whether based on transaction flows or company financial reports), and its limitations should be discussed (e.g., not covering the black market associated with crime).

- That is the issue with H2 data – nobody knows exactly how they derive their estimates, and one of the aims of the results of our paper hopefully shows this. We have highlighted this further in the results section, and also explained the limitations of this data source in the discussion section.

5.Assessment of H2 Data Reliability: 15 out of 32 studies rely on H2 Gambling Capital data, but the transparency of its methodology and the risks associated with industry interests have not been assessed.

- Indeed – this is because we cannot assess the methodology due to its lack of transparency. The same applies to many of the other studies included in this review: a proper risk of bias estimation would have required studies to be open about their methods. This was not the case for most. We have expanded on these limitations regarding the H2 methodology in the dedicated section of our results .

6.Exploration of Alternative Data Sources: Objective indicators such as bank transaction data have not been used to verify the survey results.

- You are absolutely right. We believe this relates to the overall quality of evidence in the field. We have highlighted your excellent point in the results. As none of the studies did this, we can only highlight this gap.

7.Time Window Selection Justification: The rationale for the time window selection of H2 Gambling Capital data (October 2022 to May 2024) needs to be supplemented.

- This is simply due to lack of available data prior to October 2022. We explain this in the manuscript now.

8.Unified Methodological Standards: The “Methodology” column in Table 3 needs to unify the abbreviation standards (e.g., “H2 estimate” vs. “Webtraffic analysis”); the PRISMA flowchart in Figure 1 should label the specific reasons for exclusion at each screening stage (e.g., the specific criteria for “Not Nordic”).

- You are absolutely right. We have double-checked everything and amended these.

9.Key Informant Interview Methods: The ethical review of key informant interviews (e.g., signing of informed consent forms); key informant interviews have not specified the sample size, interview guide design, and coding analysis methods.

- We expanded on this in the methodology section.

10.Explanation of Time Span Discrepancy: The literature search covers 2010-2024, but the H2 data analysis only involves 2022-2024, and the impact of the missing earlier data has not been explained.

- Yes, you are absolutely right. We only included H2 data for 2022-2024 due to data availability, as we now explain in the data section and in the results.

11.Specific Policy Recommendations: The suggestion in the conclusion to “integrate multiple data sources” needs to be supplemented with specific plans (e.g., integration pathways for bank transaction data).

- We think more work needs to go into experimenting with different data sources before we can present specific plans. However, as a general roadmap, independent scholars should first have access to different datasets to be able to evaluate them and attempt combining datasets. We have expanded on this in the discussion. We have also included other specific policy recommendations.

12.Enhanced Policy Operability: The suggestion to “combine multiple methods and datasets” has not been supported by a specific implementation framework.

- Yes, you are right. We attempted to do this in the standardized measure part of the discussion, but as outlined in our answer above, we don’t believe the current evidence is enough for us to design an implementation framework.

13.Bias Risk Assessment: Industry-funded research reports (11 out of 32 items) have not been analyzed for conflicts of interest.

- As we could not perform a proper risk of bias assessment due to lacking transparency in many reports, we have still collected data on funding to identify potential conflicts of interest. While we cannot say for certain that industry-funded reports have an inherent conflict of interest (although they very much may), we do highlight this in the results and in the discussion.

14.Analysis of Stakeholder Impact: The study mentions biases in industry reports but has not analyzed the regulatory mechanisms between regulators and operators.

- The stakeholders and regulatory mechanisms in the included countries are outside of the scope of this study. It would be very interesting, though, and would merit its own paper. We have briefly explained in the introduction hat each included country has an independent gambling regulator but that regulations to some extent vary. We have also suggested stakeholder impact as an avenue for further studies.

15.Reflection on Research Limitations: The impact of relying on secondary data on the originality of conclusions has not been reflected upon.

- We have added some reflection on this.

16.Time Window Selection Justification (Duplicate): The rationale for the time window selection of H2 Gambling Capital data (October 2022 to May 2024) needs to be supplemented.

- Done.

17.Comparative Analysis of Regulatory Effectiveness: The study should compare the regulatory effectiveness between the Nordic countries and other EU countries (e.g., the cross-border impact of Maltese licenses).

- We believe this is outside of the scope of the study, and cannot be evaluated with our dataset. However, we have referenced some prior literature indicating that the Nordic model does have some specificities in terms of regulation that may not be present elsewhere.
