# Supplementary material for: Uncertainties in measuring offshore gambling: A scoping review of Nordic approaches
Source: PLoS One. 2026 Jan 23;21(1):e0340727. doi: 10.1371/journal.pone.0340727 (PMC12829935; doi:10.1371/journal.pone.0340727)
Supplement: Attachment — Submitted filename: comments to review 2.docx [file pone.0340727.s006.docx]

1. The key informant interview section does not specify the framework for interview question design (such as a semi-structured interview outline) and lacks a description of the data analysis methods (such as thematic analysis). It is recommended to supplement the methodological details. The inclusion criteria for the “top 20 relevant results” in the screening process need to explain the rationality (such as through pre-experiment validation), and it is suggested to supplement the validation process of the screening criteria.
   - Thank you. We have included more details about the thematic interview grid and approach as well as the qualitative content analysis methods.
   - We have also explained the top 20 most relevant results in the description of the screening process.
2. The integration method of H2 data and survey data is not clearly explained (such as whether triangulation is used). It is recommended to supplement the methodological framework for data integration.
   - These methods were not integrated in the papers but presented separately. We have clarified this in the text.
3. The construction of composite measurement indicators lacks a specific technical path (such as factor analysis methods or machine learning model selection). It is suggested to supplement the methodological roadmap.
   - Our key suggestion here is that we should not rely on just the monetary values of channeling rates but also look at the population share of offshore gamblers, and the share from all gamblers. The methodological path for this is currently vague, since we do not have access to reliable data (as also described in the first point). We therefore suggest, for the time being, improving data and comparing estimates from different data sources. We will probably never achieve just one reliable figure, but many figures measuring different aspects of channelling. A composite measure was a confusing term for this, and we have revised it.
4. The channel rate charts (pages 49-51) lack a unified coordinate scale, and the comparability of data between Norway and Sweden is questionable. It is recommended to standardize the visual presentation.
   - We have standardized these figures.
5. The asterisk annotations are missing in Table 3 (such as the meaning of 93%*). It is necessary to supplement complete table annotations.
   - The annotation was at the bottom of the table but it had gotten lost on a separate page in the formatting. We clarified this.
6. The numerical alignment in Table 3 is inconsistent (such as the layout of 92%*). It is suggested to unify the numerical format.
   - Thanks we revised this.
7. The document mentions that H2 data does not cover the black market related to crime. What impact does this have on the estimation of the offshore gambling scale in Nordic countries? Are there plans to integrate alternative data sources (such as bank transaction data)?
   - To our knowledge ,in 2025, H2 estimates have started to incorporate this part of the market as well. We have updated the manuscript accordingly. We also discuss the implications of this.
8. The study points out that industry-funded reports tend to overestimate the size of the offshore market. Can the method selection (such as sample screening and expenditure multiplier assumptions) be specifically explained as to how it leads to bias? For example, is there empirical evidence for the assumption in ATG's web traffic analysis that “expenditure is 10-20 times offshore”?
   - We are not aware of any empirical evidence to support these types of claims about expenditure. The report based this estimate on information from stakeholders but did not disclose who these were or what these stakeholders based their estimates on. Industry estimates tend to be higher, and we also argue that industry tends to benefit from high estimates. We cannot know their true motivations, of course, nor why the industry reports chose to use these methods.
9. On page 36, it is proposed that reliable weights need to be allocated to different measurement methods in the composite indicators. Are there any standardized frameworks (such as entropy weight method, principal component analysis) that have been attempted? Is there a difference in weight allocation between different countries (such as Finland's monopoly system vs. Sweden's licensing system)?
   - We have revised this part of the manuscript. The research is not far enough to propose concrete ways forward towards a composite indicator and we cannot draw conclusions based on the limited evidence that we have from non-peer-reviewed reports.
10. The data sharing mechanism suggestion does not involve a specific legal framework design (such as the applicability of GDPR provisions). It is necessary to supplement the legal feasibility analysis.
    - This is an excellent point. As we are not legal scholars, we cannot do this in the current manuscript, but we have included the point in the manuscript.
11. Finland and Norway use payment blocking, but the document mentions that the black market can still bypass it through cryptocurrencies. Is there a quantitative study to assess the impact of such technical loopholes on channel rates?
    - This is an excellent question. There are no studies on this to our knowledge. We have added this in the suggestions for further studies.
12. The conclusion emphasizes the need for international cooperation to establish a unified measurement framework, but the EU lacks a coordination mechanism. What legal or technical barriers exist in the data sharing of Nordic countries (such as tax and regulatory data)?
    - We are not legal scholars and we cannot conclusively respond to this question. We have added this in the need for further studies.
13. References [73] and [76] have inconsistent DOI number formats. It is necessary to standardize the citation format.
    - We are not sure what these are – our references end with number 67?
