# Supplementary material for: Uncertainties in measuring offshore gambling: A scoping review of Nordic approaches
Source: PLoS One. 2026 Jan 23;21(1):e0340727. doi: 10.1371/journal.pone.0340727 (PMC12829935; doi:10.1371/journal.pone.0340727)
Supplement: Attachment — Submitted filename: PLOS ONE Reviewer Report.docx [file pone.0340727.s005.docx]

# ****PLOS ONE Reviewer Report****

# ****Manuscript ID:**** PONE-D-25-02743R3 ****Title:**** Measuring offshore gambling: A scoping review of Nordic approaches ****Recommendation:**** Minor revision

**Strengths**

1. The manuscript addresses a timely and policy-relevant issue in gambling regulation and public health, focusing on offshore gambling in Nordic countries.
2. The study employs a well-structured scoping review methodology, supplemented with key-informant interviews and analysis of proprietary H2 Gambling Capital data, which adds both breadth and depth.
3. The authors provide a transparent and comprehensive mapping of data sources across countries, reflecting strong familiarity with national grey literature and regulatory contexts.
4. The structure (Introduction – Methods – Results – Discussion) follows PLOS ONE guidelines, and the English writing is clear and accessible.

**Weaknesses**

1. Methodological transparency and reproducibility:

-The manuscript would benefit from more explicit details regarding screening procedures, data extraction consistency, and validation of grey-literature sources.

-The authors mention using “deductive content analysis” for key-informant interviews but do not describe the coding scheme, themes, or analytical steps.

2. Limited synthesis and comparability:

-Results are primarily descriptive. The cross-country and cross-method comparisons (e.g., between survey-, web-traffic-, and H2-based estimates) could be better summarized.

-Figures 2–5 show time trends, but without quantitative synthesis (e.g., averages, variance, or qualitative summary tables), interpretation remains difficult.

3. Interpretation and potential bias:

-The claim that “industry-funded reports tend to overestimate offshore gambling” is plausible but currently anecdotal. A clearer presentation of how these estimates differ (even qualitatively) would strengthen the argument.

-The discussion could better balance the policy implications with scientific caution, avoiding overinterpretation of motives.

**Additional Comments to the Authors**

Major revisions suggested

1. Clarify methodological steps.

Please include a short paragraph in the Methods section summarizing how study inclusion, data extraction, and verification were performed. For the key-informant interviews, add at least one example theme or coding category.

2. Summarize cross-country findings.

Consider adding a short comparative table (mean channelling rate, data source, funding type) or narrative summary to help readers grasp cross-national differences.

3. Contextualize policy discussion.

Rephrase statements implying causality or intent (“industry seeks to control the narrative”) into more cautious phrasing, such as “industry-sponsored estimates may reflect differing methodological assumptions.”

4. Provide technical clarifications.

-Add units and data sources in all figure captions.

-Specify whether H2 data trends are interpolated or derived directly from quarterly updates.

-Indicate how discrepancies between multiple estimates (e.g., from H2 vs. surveys) were handled.

**Confidential Comments to the Editor**

This manuscript is methodologically sound and policy-relevant, though descriptive. It represents one of few systematic attempts to consolidate offshore gambling measures in the Nordic context.

While the study’s originality is modest (it synthesizes existing data rather than producing new empirical results), its comprehensiveness, clarity, and applied relevance justify publication after minor revisions.

I recommend Minor Revision, primarily to enhance methodological transparency, clarify interpretive claims, and improve figure/table presentation.
