# Supplementary material for: Uncertainties in measuring offshore gambling: A scoping review of Nordic approaches
Source: PLoS One. 2026 Jan 23;21(1):e0340727. doi: 10.1371/journal.pone.0340727 (PMC12829935; doi:10.1371/journal.pone.0340727)
Supplement: Attachment — Submitted filename: comments to reviewers x.docx [file pone.0340727.s007.docx]

Thank you again to the reviewers for your thoughtful comments. We hope the paper now addresses all concerns.

Reviewer #1: The revisions of the manuscript are inadequate.
The research lacks the requisite data validation, and the credibility of the conclusions is insufficient.

- As explained in the manuscript, the point of this paper is to investigate the shaky evidence base that underlies significant political decisions (and lobbying). Channelling rates are extensively used by industry to lobby against regulations and. Yet, as we show in our scoping review, much of the evidence in this field is produced by industry and using methods that are not open nor always credible.
- We cannot conduct the requisite data validation precisely for this purpose: the data underlying channelling debates is not openly available and the methods cannot usually be thoroughly investigated.
- We have further highlighted the aims of our study in the introduction and discussion. We hope this is clearer now.

Meanwhile, it is recommended that the structure of the manuscript should be re-organized.

- We have read through the manuscript carefully and reorganized the text where this seemed fitting. We were not sure what part this comment referred to.

Reviewer #6: 1.Add Details on Key Informant Interviews. In the methods section, the criteria for selecting interviewees should be clearly stated (e.g., job level, professional field). The principles for designing the interview outline should also be described, as well as the qualitative analysis method used (e.g., framework analysis or thematic analysis).

- Thank you, this is a good comment. We have substantially improved the section on the key informant data collection and analysis.

2.Add H2 Data Validation Process:。The reliability of the H2 gambling capital data should be verified. This includes methods for cross - validation with other data sources, strategies for seasonal adjustment of time - series data, and specific criteria for classifying gray/black market data.

- We would love to do this, but it is unfortunately impossible. As we explain in the introduction, this paper aims at investigating the uncertainties involved in measuring the offshore market – not producing a reliable measure of the market.
- We cannot reliably cross-valdiate the H2 data because the other data points are equally questionable, using differing methodologies and assumptions that underly an field that is largely captured by industry interests rather than rigorous methodological development.
- Even measures by H2 vary in time, making it impossible to compare the evolution of channelling rates in time or across each other.
- We have tried to further emphasise in the text that the paper focuses on uncertainties in existing measures rather than attempting to synthesise measures that are, on the basis, unreliable and often produced for political rather than scientific purposes.

Reviewer #8: While the topic is timely and the regional focus is well justified, the manuscript reads largely as a descriptive policy review rather than an analytical scientific study. The methodological transparency is limited, the synthesis remains largely narrative, and the discussion offers little conceptual advancement beyond the existing literature. The paper would benefit from clearer methodological framing, more critical comparison across data sources, and a deeper analytical discussion.

- Thanks. This is a big comment but we have tried to address this in the manuscript by highlighting that our manuscript is a scoping study focusing on the epistemic uncertainty of this specific field of study. Our study outlines some constituents of this epistemic situation, e.g. the intransparent of data sources and general data opacity, data proxies, industry self-reports.
- Our study does not aim at synthesizing ‘neutral’ observations on the size of offshore gambling. Rather, drawing from critical epistemology, we explore the characteristics and uncertainties that relate to the layers involved in this field. Across the Nordics, different regulatory regimes set the need to measure the unlicensed gambling market, but there are no gold standards and all measurements are constrained by the same epistemic uncertainty.
- This framing is now made more explicit in the introduction and in the discussion.
