# Supplementary material for: Uncertainties in measuring offshore gambling: A scoping review of Nordic approaches
Source: PLoS One. 2026 Jan 23;21(1):e0340727. doi: 10.1371/journal.pone.0340727 (PMC12829935; doi:10.1371/journal.pone.0340727)
Supplement: Attachment — Submitted filename: PLOS ONE response to reviewer x.docx [file pone.0340727.s008.docx]

**Strengths**

1. The manuscript addresses a timely and policy-relevant issue in gambling regulation and public health, focusing on offshore gambling in Nordic countries.
2. The study employs a well-structured scoping review methodology, supplemented with key-informant interviews and analysis of proprietary H2 Gambling Capital data, which adds both breadth and depth.
3. The authors provide a transparent and comprehensive mapping of data sources across countries, reflecting strong familiarity with national grey literature and regulatory contexts.
4. The structure (Introduction – Methods – Results – Discussion) follows PLOS ONE guidelines, and the English writing is clear and accessible.

- Thank you for your comments – we hope the paper would now be fit for publication.

**Weaknesses**

1. Methodological transparency and reproducibility:

-The manuscript would benefit from more explicit details regarding screening procedures, data extraction consistency, and validation of grey-literature sources.

-The authors mention using “deductive content analysis” for key-informant interviews but do not describe the coding scheme, themes, or analytical steps.

- Thanks for this comment – as explained during the previous review round, we cannot validate these data since these data are not reliable in the first place – the aim of the manuscript is to investigate the uncertainties and variability of measures in this field. We have emphasized this in the methods section.
- We have expanded on the description of the coding scheme for the qualitative key informant interviews.

2. Limited synthesis and comparability:

-Results are primarily descriptive. The cross-country and cross-method comparisons (e.g., between survey-, web-traffic-, and H2-based estimates) could be better summarized.

-Figures 2–5 show time trends, but without quantitative synthesis (e.g., averages, variance, or qualitative summary tables), interpretation remains difficult.

- Thanks! The results are descriptive because this is the first scoping study within this field. We have added a clear summary of the differences across methods in the results.
- We believe that Figures 2-5 should not have a quantitative synthesis because the aim here is to showcase differences in estimates across years and between operator / other estimates. Adding quantitative synthesis would be relevant if the figures were trustworthy – which, as we argue in this paper, they are not.

3. Interpretation and potential bias:

-The claim that “industry-funded reports tend to overestimate offshore gambling” is plausible but currently anecdotal. A clearer presentation of how these estimates differ (even qualitatively) would strengthen the argument.

-The discussion could better balance the policy implications with scientific caution, avoiding overinterpretation of motives.

- These are both very good points. We have also added a few sentences to clarify how industry and non-industry estimates differ.
- We have also revised the discussion to avoid words and expressions that might suggest bias.

**Additional Comments to the Authors**

Major revisions suggested

1. Clarify methodological steps.

Please include a short paragraph in the Methods section summarizing how study inclusion, data extraction, and verification were performed. For the key-informant interviews, add at least one example theme or coding category.

- Thanks we have done these.

2. Summarize cross-country findings.

Consider adding a short comparative table (mean channelling rate, data source, funding type) or narrative summary to help readers grasp cross-national differences.

- We do not want to provide a summary table with mean channelling rates because this would suggest that estimates are reliable – which they are not. In addition, there is so much change between years that this would not tell much. Instead, we have drafted a paragraph where we draw together all the comparative aspects.

3. Contextualize policy discussion.

Rephrase statements implying causality or intent (“industry seeks to control the narrative”) into more cautious phrasing, such as “industry-sponsored estimates may reflect differing methodological assumptions.”

- Thanks, we have revised the language in the discussion to be more cautions. However, we did not change this particular phrase as it came from our key informants.

4. Provide technical clarifications.

-Add units and data sources in all figure captions.

-Specify whether H2 data trends are interpolated or derived directly from quarterly updates.

-Indicate how discrepancies between multiple estimates (e.g., from H2 vs. surveys) were handled.

- We went through all the annotations and captions of our figures but we fail to see where we should add further information – all figures and tables are derived from our analysis and use channelling rate as the unit. This is explained in the text.
- H2 figures are just estimates given at certain period (or if they stayed the same). H2 doesn’t have any “regular update schedule”, they update the numbers if they think they have changed somehow (or their new estimate is “more precise”). We have specified this in the appropriate section dealing with H2 updates.
- The H2 numbers and survey numbers measure completely different things so these cannot really be summarized together. We have highlighted this in the results and methods sections.
